# Effects of cadmium stress on fruits germination and growth of two herbage species

**DOI:** 10.1515/biol-2022-0544

**Published:** 2023-04-11

**Authors:** Ying Hu, Huichun Wang, Biyao Zhou, Zhengke Li, Huiping Jia, Pengmao Deji, Nian Liu, Jingjing Wei

**Affiliations:** College of Life Sciences, Qinghai Normal University, Xi’ning 810008, China; Key Lab. of Medicinal Animal and Plant Resources on the Qinghai–Tibet Plateau, Xi’ning 810008, China; The south of Qilian Mountain Forest Ecosystem Observation and Research Station, Huzhu 810500, China; College of Geographical Sciences, Qinghai Normal University, Xi’ning 810008, China; Qinghai Province Ecological Environment Monitoring Center, Xi’Ning 810007, China

**Keywords:** Tibetan Plateau, cadmium stress, fruits germination, fruits growth, *Elymus sinosubmuticus*, *Elymus tangutorum*

## Abstract

Cadmium (Cd) pollution is a global environmental problem. It is of great significance to find a kind of pasture that can grow normally in a cadmium environment, especially in the Tibetan Plateau. We studied the fruit germination and fruit growth of *Elymus sinsubmuticus* S.L. Chen and *Elymus tangutorum* (Nevski), native plants of the Tibetan Plateau, in different cadmium environments. The results showed that with increased cadmium stress, the fruit germination rate, final germination rate, fruit-vigor, average germination time, and germination-speed index for the two grass species gradually decreased, and the 50% germination time for the seed gradually increased. Root length, biomass, and the number of leaves decreased in both species. We quantified the fruit germination and growth of plants in the cadmium environment and found that *E. sinosubmuticus* S.L. Chen had better fruit germination and fruit growth, and it had the development potential of cadmium pollution control.

## 1 Introduction

In recent years, environmental issues have become topics of discussion among scientists worldwide. Faced with these challenges, we need to study the existential risks posed by environmental pollution from more perspectives [1,2]. Cadmium is a metal used in the manufacture of products such as alloys, dyes, plastics, pesticides, paints, nickel–cadmium, silver–cadmium, and lithium–cadmium rechargeable batteries [3]. The production and sale of these commodities cover almost all human activities, such as industry, agriculture, mining, and transportation [4]. Through commercial wastes, cadmium can enter the air, water, and soil, causing environmental pollution [5,6]. As a nonessential nutrient for plants, cadmium is mainly absorbed into the plant body through the plant roots, resulting in the reduction of chlorophyll in plant leaves, the increase in antioxidant enzymes, the inhibition of plant growth, and even plant death [7]. At the same time, as the primary producers, the cadmium in plant matter causes cadmium poisoning in other organisms, along with an extension to the food chain and cadmium’s transmission in the food web, becoming a threat to the health of humans [8].

Fruits are the common propagule of gymnosperms and angiosperms, which ensure the reproduction and renewal of sexually reproductive plants [9]. Cadmium exposure can have an impact on plant-fruit germination, altering fruit germination rates and fruit growth. Studies have found that under cadmium stress, pea-fruits’ germination radicle and embryo growth are significantly inhibited, and the hydrolysis of the main storage proteins (albumin, Leguminosae, and Westwood) is damaged. This results in a reduced release of free amino acids (cysteine, aspartic acid, and serine) and a lack of nitrogen supply, an essential nutrient for pea seed growth. At the same time, oxidative stress and excess free radicals are produced [10]. Cadmium reduces bean-fruit germination rates, embryo growth, biomass, and mineral and sugar partitioning between cotyledons and hypocotyls, inhibits α-amylase and invertase (soluble acid, soluble neutral, and cell-wall-bound acid) activity. The content of malondialdehyde and the activity of lipoxygenase are increased, and the delayed intensity of fruit germination increases gradually with the increase in cadmium concentration [11]. With the gradual increase in cadmium concentration, the root length, stem length, biomass (root and stem), chlorophyll content, and stomatal conductance of quinoa all decrease, and the activities of antioxidant enzymes (superoxide dismutase, catalase, peroxidase) gradually increase [12].

Cadmium pollution is a global environmental problem. The Yamuna River, which originates in the Himalayas, is one of the main tributaries of the Ganges River in India. The contents of Fe, Cu, Co, Zn, Pb, Ni, and Cr in the river water are 40–190, 50–120, 4–66, 840–1,800, 2–40, 100–600, 88–253, and 35–52 μg L^−1^ [13]. As early as 1999, heavy metal elements, such as Pb, Cu, Zn, Cd, As, Sb, and Br, were detected by soil analysis in the Himalayan Mountains, where the Yamuna River originates. These elements can not only come from the local soil but also be derived from other natural or anthropogenic sources through *tangutorum* migration [14]. Of Poland’s 23 national parks, the Babiogórski, Ojcowski, Gorczański national parks in the south of the country are the most heavily contaminated by heavy metals [15]. Cd, Cu, and Pb are the main pollutants in the surface sediments from the upper reaches of the Red River in Vietnam down to the underwater delta, and their concentrations reach moderate-to-severe pollution levels [16]. From a global perspective, transportation is a typical source of heavy-metal pollution. There are different concentrations of heavy metals in urban road dust in Asia, Europe, Africa, North and South America, and Australia. The zinc content in the road dust of Europe is the highest, followed by Asia, Africa, Australia, and America. The Cu pollution and pollution-load index (PLI) are highest in Europe and lowest in Africa. The PLI in the USA and African cities is between the two. The potential ecological risk of different continents is highest in Asia, followed by Europe, Australia, America, and Africa [17].

The Qinghai–Tibet Plateau is recognized as the “Third Pole” and the “Water Tower of Asia” [18]. The highest and largest alpine grassland in the world is in the Plateau [19]. The grassland-dominated ecosystem covers an area of 2.5 × 10^6^ km^2^, accounting for more than 60% of the total plateau area; grassland resources are valuable for the survival of local herdsmen and many wild animals [20] and are also an important component of fragile ecological barriers [21]. However, heavy metal pollution has gradually appeared in the Qinghai–Tibet Plateau. The Huangshui Watershed is located in the eastern part of the Qinghai–Tibet Plateau. The average concentrations of heavy metals in surface and deeper soils are 124.85 and 104.10 mg kg^−1^ for Cr, 0.36 and 0.23 mg kg^−1^ for Cd, 34.07 and 28.99 mg kg^−1^ for Cu, 35.23 and 28.43 mg kg^−1^ for Pb, and 106.16 and 92.26 mg kg^−1^ for Zn, respectively [22]. Highways in the eastern part of the Tibetan Plateau are contaminated with heavy metals to varying degrees. The road segments stretch from Xining to Maduo along the #214 national highway, from Maduo to Qumalai, from Qumalai to Budongquan off the #308 provincial highway, from Budongquan to Naqu off the #109 national highway, and from Naqu to Lhasa off the #109 national highway in the Qinghai–Tibet Plateau. The mean concentrations of Cu (23.13 mg kg^−1^), Zn (100.97 mg kg^−1^), Cd (0.30 mg kg^−1^), Pb (29.60 mg kg^−1^), Ni (31.93 mg kg^−1^), and As (21.40 mg kg^−1^) of the entire region were higher than the background values, and the concentrations of Cr (36.20 mg kg^−1^) and Co (10.31 mg kg^−1^) were lower than the background values [23]. In the main industrial, mining, and agricultural areas and transportation lines in the northeastern part of the Qinghai–Tibet Plateau, the mean values of Hg and Cd were 0.28 and 0.68 mg kg^−1^, respectively, and the highest concentrations were 0.80 and 14.84 mg kg^−1^, respectively. The maximum concentrations of Hg and Cd in the soil were 40 and 108 times larger than the background values, respectively. Among all the heavy metals, lead had the highest concentration, which was 2076.28 mg kg^−1^. The average concentrations of Zn, Cr, and V were 145.64, 93.29, and 83.10 mg kg^−1^, respectively [24]. Cadmium pollution on the Qinghai–Tibet Plateau mainly comes from industry, transportation, landfills, and atmospheric transmission [3,25,26].

Although metal concentrations are well characterized, there is little knowledge about the fruit germination and fruit growth of alpine plants under cadmium stress. This article is the first to report on fruit germination and fruit growth under cadmium stress for *Elymus sinosubmuticus* S.L. Chen and *Elymus tangutorum* (Nevski) Hand.-Mazz., which originate in the Qinghai–Tibet Plateau. We aim to clarify the growth situation of alpine plants in response to the increased risk of cadmium pollution. At the same time, to compensate for the scarcity of phytoremediation species available for alpine heavy metals, we selected and then comprehensively evaluated these two grass species for their superior phytoremediation capability and cadmium resistance.

## 2 Materials and methods

### 2.1 Experimental design

*Elymus sinosubmuticus* S.L. Chen (*Els*) and *E. tangutorum* (Nevski) Hand.-Mazz. (*Elt*) were grown by Tongde Feed Fruits Production in Qinghai Province, China (35°15′N, 100°38′E), which is located in the eastern part of the Qinghai–Tibet Plateau [27]. The soil pH in this area is about 7–8, and the soil background value of cadmium is less than 0.2 mg L^−1^ (Environmental Quality Standard for Soils of China, GB15618-1995). Healthy and plump fruits were selected, sterilized with 10% (v/v) H_2_O_2_ for 30 min, rinsed with distilled water three times [28], and then soaked in distilled water at 25°C for 24 h. After the filter paper absorbed the surface moisture, the fruits were evenly dispersed in a culture dish with a diameter of 9 cm; 50 fruits were placed in each culture dish; three filter papers were laid in the culture dish in advance; and 5 mL of different concentrations of cadmium-stress solution (0, 10, 20, 30, 40, 50 mg L^−1^) was added (the stress solution was prepared with distilled water). There were three repeated treatments for each concentration. Simulations were carried out under day (25 ± 2°C, 12 h 5,000 lx light, PAR 400–700 nm, 70% humidity) and night (20 ± 2°C, 12 h 0 lx dark, 60% humidity) conditions, in an artificial intelligence incubator. The fruits were carefully cultivated in the medium, and a corresponding stress solution was regularly added to maintain the humidity and stress balance of the medium. The germination of the fruits was recorded from the third day; when the radicles of the fruits were more than half the fruits’ length, this was regarded as germination. The seedlings were cultivated for 23 days, and the test data were recorded (the stress solution was prepared with Hoagland plant nutrient solution during seedling cultivation).

### 2.2 Index measurement and data calculation

Fruits germination rate (Gi), final germination rate (Gf), germination-speed index (GSI), and 50% germination time (*T*_50_) were calculated with reference to the study of Sneideris et al. [29].

In Formula (2), Gi represents the germination rate of seeds, *X*_*i*_ represents the number of seeds germinated in consecutive *i* days, and *N* represents the total number of experimental seeds.

(1) {\rm{Gi}}=\frac{{X}_{i}}{N}\times 100 \% ,]

(2) {\rm{Gf}}=\frac{{n}_{f}}{N}\times 100 \% .]

In Formula (2), Gf represents the final germination rate and *n*_*f*_ represents the number of fruits germinated at the end of the whole experiment (14 days for Elymus and 16 days for Palladium).

(3) {\rm{GSI}}=\sum \frac{{n}_{i}}{{t}_{i}}.]

In Formula (3), GSI represents the germination-speed index, *ni* represents the number of fruits germinated on the *i*th day, and *t*_*i*_ represents the *i*th day.

(4) {T}_{50}={t}_{i}+\frac{(\frac{N}{2}\left-{n}_{i})\left\times ({t}_{j}\left-{t}_{i})}{{n}_{j}-{n}_{i}}.]

In Formula (4), *T*_50_ represents 50% germination time, *N* is the number of final germination fruits, and *n*_*i*_ and *n*_*j*_ are the cumulative numbers of germination fruits counted at adjacent *t*_*i*_ and *t*_*j*_ moments when *n*_*i*_ < *N*/2 < *n*_*j*_ (the experiment was calculated according to *Els* of 6–14 days and *Elt* of 3–16 days).

The fruits-vigor index (VI) and mean germination time (MGT) were calculated according to the research calculation method of Nabaei and Amooaghaie [30]:

(5) {\rm{VI}}={\rm{SL}}\times {\rm{Gf}}.]

In Formula (5), VI is the fruits-vigor index, SL is the fruits length, and Gf is the final germination rate.

(6) {\rm{MGT}}=\frac{{G}_{t}{D}_{t}\hspace{1em}}{G}.]

In the Formula (6), MGT represents the average germination time, *G*_*t*_ represents the number of fruits germinated on *t* day, *D*_*t*_ represents the *t* day, and *G* is the number of fruits germinated (the experiment was calculated over 20 days).

We continued to cultivate the plants for 23 days, recorded the number of leaves (Ln) of the fruits, measured the root length (Rl) and stem–leaf length (Gh) of fruits with a millimeter vernier caliper, and measured the root weight (Rw), fruits weight (Lw), and biomass (Bi) of fruits by electronic analysis balance. In accordance with the research of Fang et al. [31], the membership-function index method was used to calculate the cadmium tolerance of two plant fruits:

(7) X({\mu })=\frac{X-{X}_{{\rm{\min }}}}{{X}_{{\rm{\max }}}-{X}_{{\rm{\min }}}}]

(8) \bar{{X}_{j}}=\frac{1}{n}\mathop{\sum }\limits_{i=1}^{n}{X}_{{ij}},]

(9) {V}_{j}=\frac{\sqrt{{\sum }_{i=1}^{n}{({X}_{{ij}}-\bar{{X}_{j}})}^{2}}}{\bar{{X}_{j}}},]

(10) {W}_{j}=\frac{{V}_{j}}{\mathop{\sum }\limits_{j=1}^{m}{V}_{j}},]

(11) D=\mathop{\sum }\limits_{j=1}^{n}{[}\mu ({X}_{j}){\rm{\cdot }}{W}_{j}].]

First, indicator data were normalized using a subordinate function. Here, *X*(*μ*) is the membership function value of the *μ*th index, *X* is the average value of the evaluation index, *X*_max_ is the maximum value of the evaluation index, and *X*_min_ is the minimum value. *X*_*j*_ is the average value of the *j*th evaluation index, *n* is the number of varieties, and *X*_*ij*_ is the *j*th evaluation index for the *i*th variety. *V*_*j*_ is the standard deviation coefficient of the *j*th evaluation index, and *X*_*j*_ is the *j*th evaluation index for the variety. *W*_*j*_ is the weighting coefficient of the *j*th evaluation index, and *μ*(*X*_*j*_) is the membership function value of the evaluation index. *D* is the comprehensive value of planting cadmium tolerance under Cd stress. The larger the *D* value, the higher the Cd tolerance of the plant variety.

### 2.3 Statistical approach

Microsoft Excel 2019 software was used for data sorting and statistics. Shapiro–Wilk (S–W) test for normal distribution. One-way analysis of variance (ANOVA) was used to analyze the significance of the difference between fruit germination and fruit growth, and the least significance difference test was used for multiple comparisons among multiple sample means. The significance test of different treatments was obtained by using the VARIANCE analysis in SPSS 17.0 (SPSS Inc., Chicago, IL, USA); *p* < 0.05 indicated a significant difference. The Origin 2022 (OriginLab Corporation, Northampton, MA, USA) software was applied to complete the trend chart and correlation analysis chart.

## 3 Results

### 3.1 Effects of cadmium stress on fruit germination of *Els* and *Elt*

**Figure 1:**
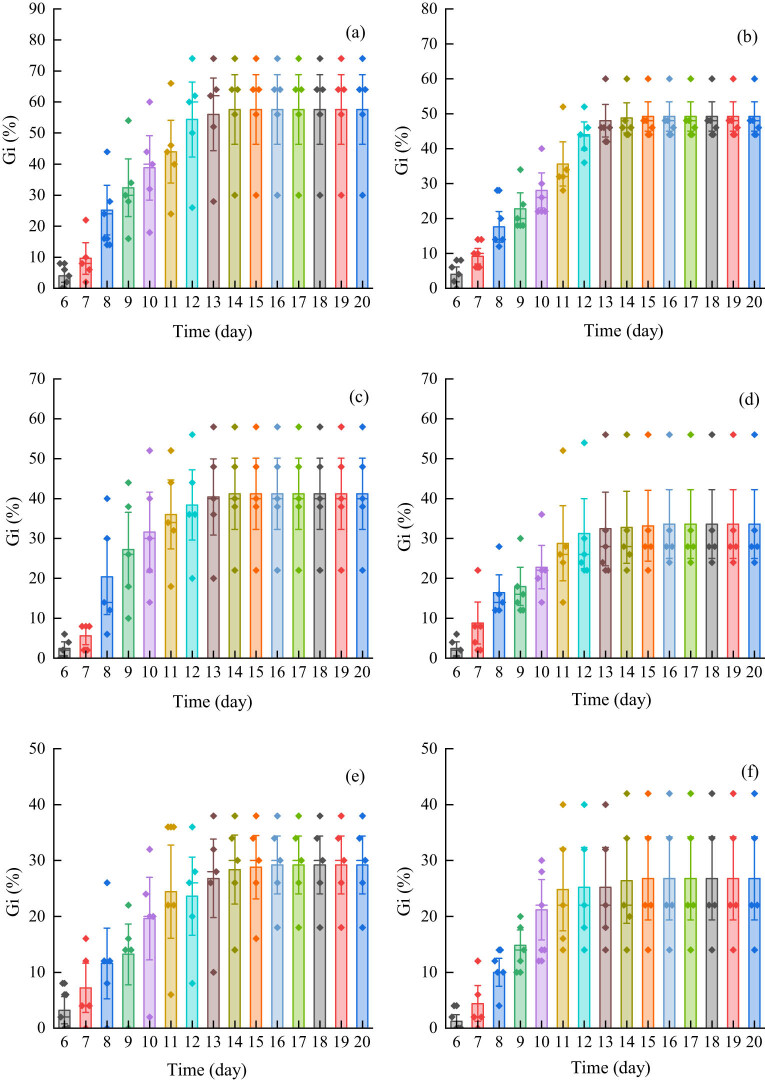
Fruit germination rate for *E. sinosubmuticus* S.L. Chen under cadmium stress, *p* < 0.05: (a) blank control group; (b) 10 mg L^−1^ treatment group; (c) 20 mg L^−1^ treatment group; (d) 30 mg L^−1^ treatment group; (e) 40 mg L^−1^ treatment group; and (f) 50 mg L^−1^ treatment group. The different colored diamonds represent the specific data distribution for each group. Each column represents the cumulative germination rate of the plant.

**Figure 2:**
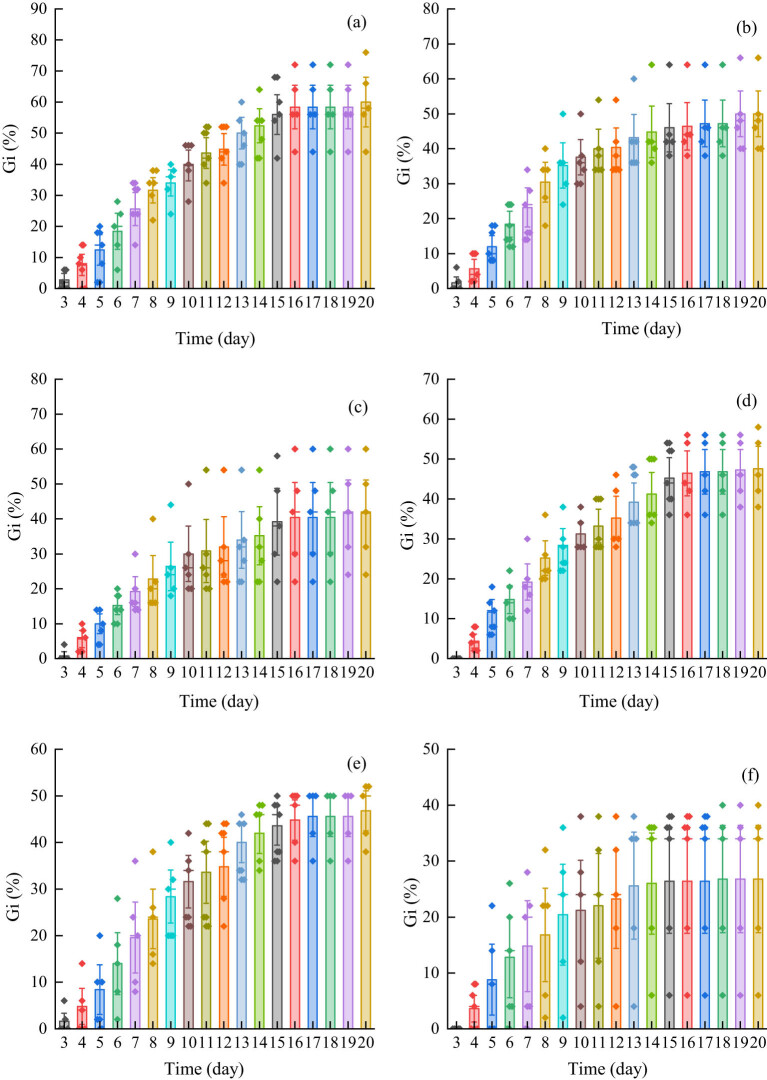
Fruit germination rate for *E. tangutorum* (Nevski) Hand.-Mazz. under cadmium stress, *p* < 0.05: (a) blank control group; (b) 10 mg L^−1^ treatment group; (c) 20 mg L^−1^ treatment group; (d) 30 mg L^−1^ treatment group; (e) 40 mg L^−1^ treatment group; and (f) 50 mg L^−1^ treatment group. The different colored diamonds represent the specific data distribution for each group. Each column represents the cumulative germination rate of the plant.

Under cadmium stress, the germination rate for the fruits of *Els* increased gradually and reached the maximum germination rate on the 14th day in each treatment group (Figure 1). The germination rate for *Elt* fruits also increased gradually and reached the maximum germination rate on the 16th day in each treatment group (Figure 2). The results indicated that the cadmium treatment had no effect on the maximum germination time for the two kinds of herbage.

**Figure 3:**
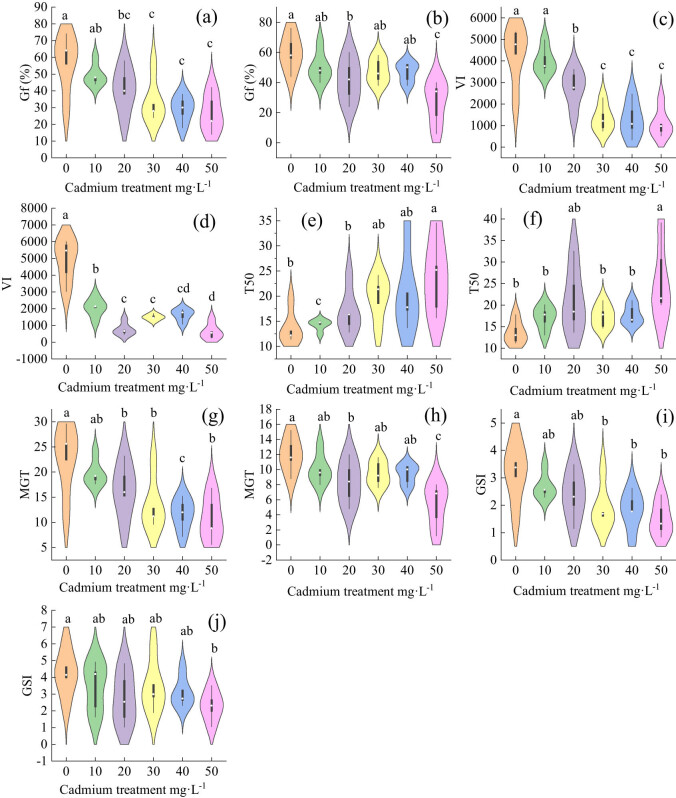
Fruit germination of two grass species under cadmium stress (both significant, *p* < 0.05): (a) final germination rate for *E. sinosubmuticus* S.L. Chen; (b) final germination rate for *E. tangutorum* (Nevski) Hand.-Mazz.; (c) *E. sinosubmuticus* S.L. fruits vigor; (d) *Elt* fruits vigor; (e) 50% germination time for *E. sinosubmuticus* S.L. Chen; (f) 50% germination time for *E. tangutorum* (Nevski) Hand.-Mazz.; (g) average germination time for *E. sinosubmuticus* S.L. Chen; (h) average germination time for *E. tangutorum* (Nevski) Hand.-Mazz.; (i) GSI for *E. sinosubmuticus* S.L. Chen; and (j) GSI for *E. tangutorum* (Nevski) Hand.-Mazz.

Compared with the control group, the final germination rate for *Els* was significantly different after the treatment level of 30 mg L^−1^, and it decreased with the increase in cadmium concentration (Figure 3a). There was a significant difference in the final germination rate at the 50 mg L^−1^ treatment level of *Elt*, and the change in the final germination rate was consistent with that of *Els*, but the trend was not obvious (Figure 3b).

Compared with the control group, the cadmium stress significantly reduced the fruits vigor of *Els*. Under the stress concentration of 10–30 mg L^−1^, the fruits vigor decreased significantly with the increase in cadmium stress. However, when the stress concentration was greater than 30 mg L^−1^, there was no significant change in fruits vigor with the increase in cadmium concentration (Figure 3c). The decreasing trend was not obvious when the stress concentration exceeded 20 mg L^−1^ (Figure 3d).

Cadmium stress could increase the germination time for *Els* fruits by 50%, and the time increased with the increase in cadmium concentration. When the stress concentration exceeded 30 mg L^−1^, the difference in effects between treatments was not obvious (Figure 3e). The cadmium had little effect on the germination time for 50% of the *Elt* fruits, and it showed a significant effect when the stress concentration exceeded 50 mg L^−1^ (Figure 3f).

The cadmium stress significantly reduced the average fruit germination time for *Els*, when the stress concentration was greater than 20 mg L^−1^ and the inhibition effect was very obvious (Figure 3g). The change in the average fruit germination time for *Elt* under cadmium stress was the same as that for *Els* (Figure 3h).

In the fruit-germination-rate index, both plants showed a decreasing trend with the increase in cadmium concentration. The decreasing trend of *Els* was more significant than that for *Elt*, when the stress concentration was greater than 30 mg L^−1^, and the treatment effect between the treatment group and the illuminance was significantly stronger (Figure 3i), but this situation was only observed when the grass was treated at 50 mg L^−1^ (Figure 3j).

### 3.2 Effects of cadmium stress on fruits growth of *Els* and *Elt*

**Figure 4:**
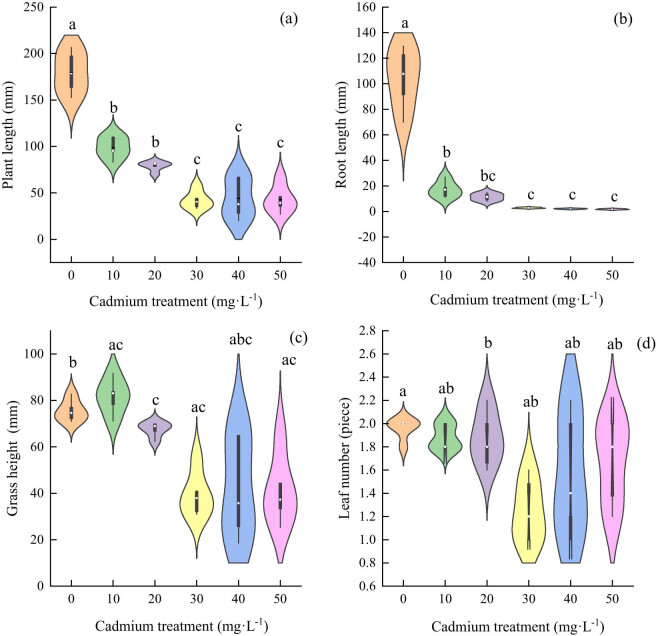
The effect of cadmium stress on the growth of *E. sinosubmuticus* S.L. Chen: (a) total plant length of each treatment level, *p* < 0.05; (b) root length of each treatment level, *p* < 0.05; (c) lengths of stems and leaves of the plants in each treatment level, *p* < 0.05; and (d) number of leaves of the plants in each treatment level, *p* < 0.05.

Under cadmium stress, the plant height of each treatment group was significantly different from that of the control group, and decreased with the increase in the treatment concentration (Figure 4a). Compared with the control group, the root length in the cadmium-treatment group was significantly inhibited, and there was no significant difference among the treatment groups (Figure 4b); the heights of the stems and leaves of each treatment group were different from those of the control group, which decreased with increasing stress concentration (Figure 4c); there was no significant difference in the number of leaves between the treatment groups and the control group (Figure 4d). The results showed that cadmium stress inhibited plant height, root length and stem length of *Els*.

**Figure 5:**
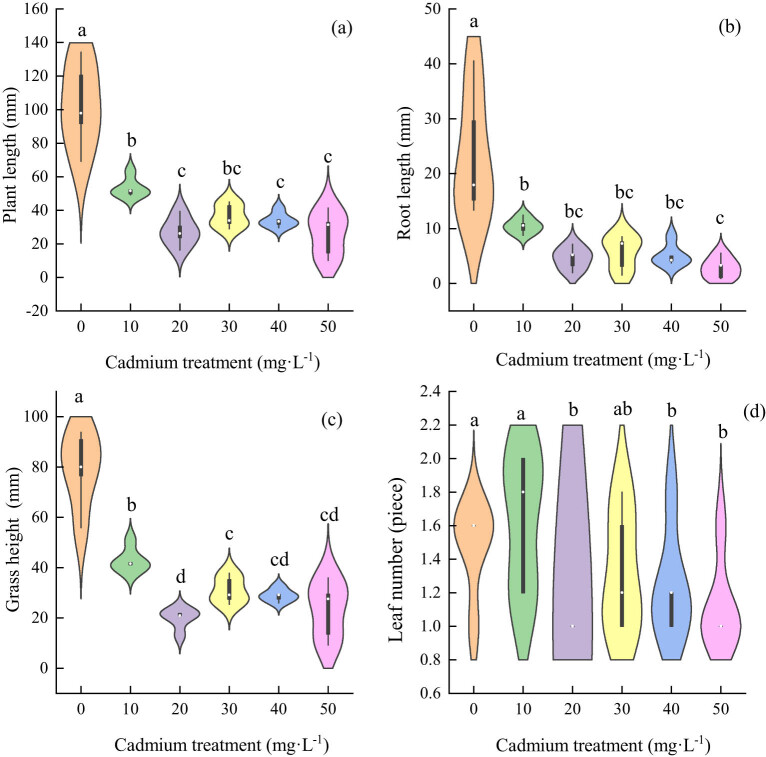
The effect of cadmium stress on the growth of *E. tangutorum* (Nevski) Hand.-Mazz.: (a) total plant length of each treatment level, *p* < 0.05; (b) root length of each treatment level, *p* < 0.05; (c) length of stem and leaf in each treatment level, *p* < 0.05; and (d) number of leaves in each treatment level, *p* < 0.05.

In the growth situation of *Elt* under the cadmium stress, the difference in the total plant length between each treatment group and the control group was extremely significant. The plant height decreased with the increase in cadmium concentration (Figure 5a). The difference in root length between all the treatment groups and the control group was extremely significant, but the difference between treatments was not significant and decreased with the increase in stress concentration (Figure 5b). The stem and leaf lengths of each treatment group and the control group were significantly different, but the difference between treatments was not significant and decreased first and then increased with the increase in stress concentration (Figure 5c). The number of leaves in all treatment groups decreased with the increase in cadmium concentration (Figure 5d). The results showed that the cadmium stress also inhibited fruit growth.

**Figure 6:**
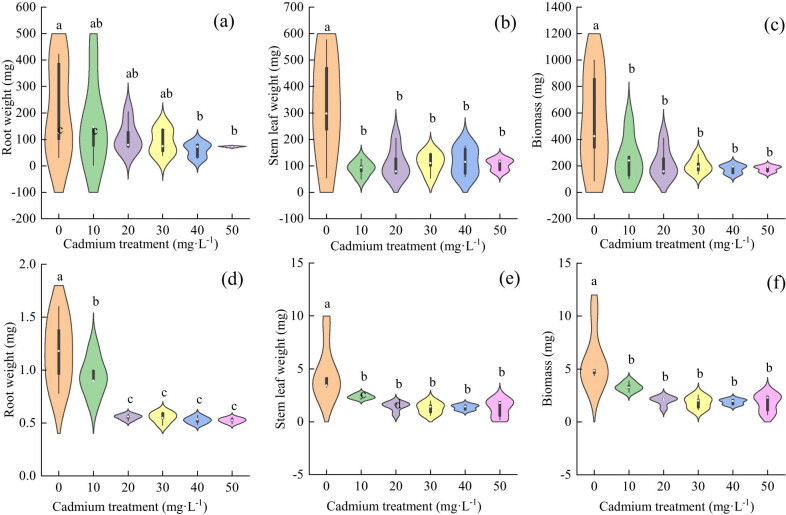
The effect of cadmium stress on the biomass of two grass species; both were significant *p* < 0.05: (a) root weight for each treatment level of *E. sinosubmuticus* S.L. Chen; (b) stem and leaf biomass for each treatment level of *E. sinosubmuticus* S.L. Chen; (c) total plant biomass for each treatment level of *E. sinosubmuticus* S.L. Chen; (d) root weight for each treatment level of *E. tangutorum* (Nevski) Hand.-Mazz.; (e) total plant biomass for each treatment level of *E. tangutorum* (Nevski) Hand.-Mazz.; and (f) biomass of plant stems and leaves for each treatment level of *E. tangutorum* (Nevski) Hand.-Mazz.

With the increase in cadmium concentration, the root biomass of *Els* decreased gradually, and there was a significant difference between the control group and the 40 mg L^−1^ treatment group (Figure 6a); there was a significant difference in stem and leaf biomass between the control and treatment groups; there was no significant difference between the treatment groups (Figure 6b); and the total biomass was consistent with the changes in stem and leaf biomass (Figure 6c). The root biomass of *Elt* decreased gradually. When the stress concentration was greater than 20 mg L^−1^, there was no significant difference between the treatment groups (Figure 6d). The change rules for stem and leaf biomass and total biomass were consistent. The biomass of the groups was inhibited, and there was no significant difference among the treatment groups (Figure 6e and f).

**Figure 7:**
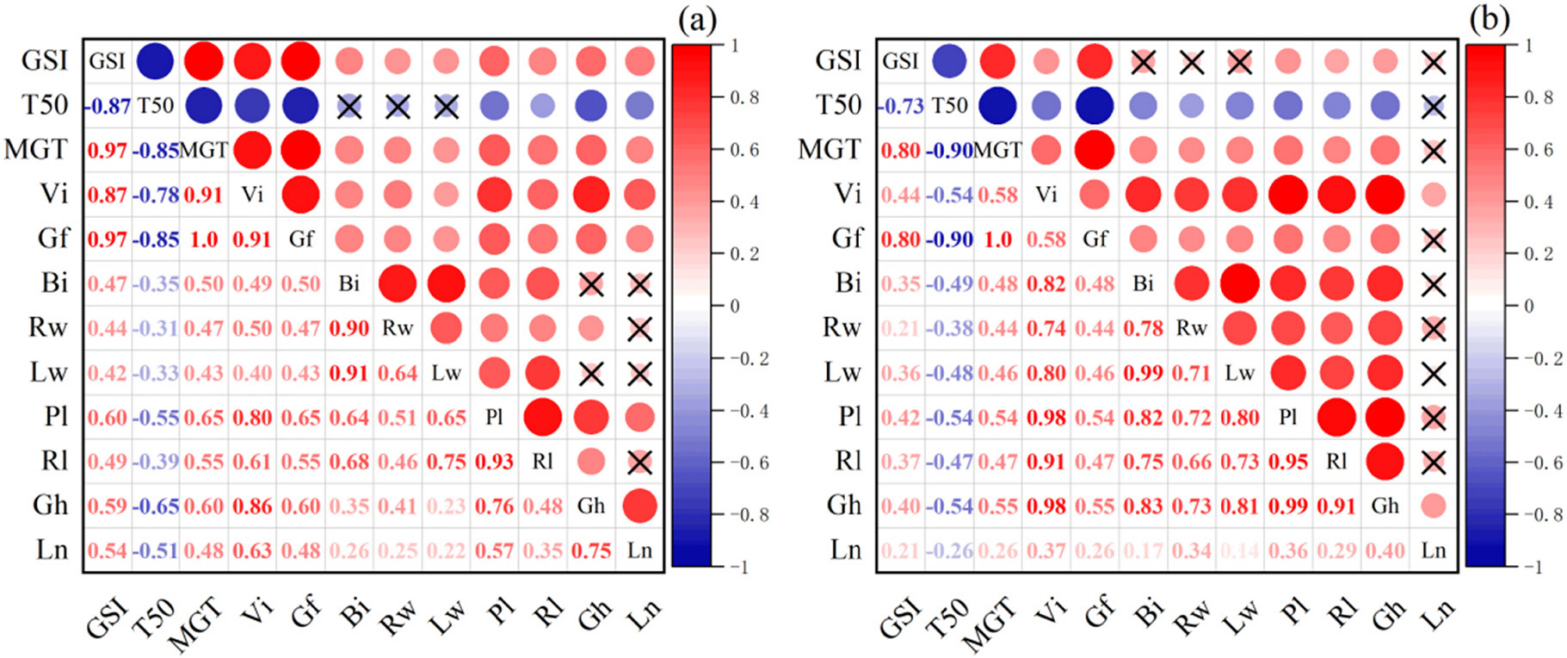
Pearson correlation analysis of each index under cadmium stress; the significance level is 0.05: (a) *E. sinosubmuticus* S.L. Chen and (b) *E. tangutorum* (Nevski) Hand.-Mazz.

Under cadmium stress, the *T*_50_ for *Els* was negatively correlated with GSI, MGT, VI, and Gf; in the positive correlation, the correlation coefficients between GSI, MGT, VI, and Gf were all greater than 0.85, and the correlation coefficients between MGT, VI, and Gf were also different. When reaching 0.91 and 1.0, the correlation coefficient between VI and Gf was 0.91, and their correlation was strong; in the fruit growth indicators, the correlation coefficients between Bi and Rw and Lw were 0.90 and 0.91, and the correlation between Pl and Rl was 0.93. These indicators were linked to each other and were very strong (Figure 7a). In the positive correlation, the results were as follows: the correlation coefficient between GSI and MGT and Gf was 0.80; the correlation coefficient between MGT and Gf was 0.80 and 1.0; the correlation coefficients between VI and Gh, Rl, Pl, Lw, Rw, Bi, and Gf were 0.98, 0.91, 0.98, 0.80, 0.74, and 0.82, respectively; the correlation coefficients between Bi and Gh, Rl, Pl, Lw, and Rw were 0.83, 0.75, 0.82, 0.99, and 0.78, respectively; the correlation coefficients between Pl and Gh and Rl were 0.99 and 0.95, respectively; and the correlation coefficient between Rl and Gh was 0.91, indicating strong correlation.

### 3.3 Evaluation of cadmium tolerance in *Els* and *Elt*

Under cadmium stress, the *D* value of *Els* was 3.79, which was higher than that of *Elt*, indicating that *Els* was more tolerant to the cadmium than *Elt* in terms of fruit germination and fruit growth (Table 1).

**Table 1:** Comprehensive evaluation of fruit germination and fruit growth of *E. sinosubmuticus* S.L. Chen and *E. tangutorum* (Nevski) Hand.-Mazz

Species	Index																									
	Cd	GSI		*T*_50_		MGT		VI		Gf		Bi		Rw		Lw		Pl		Rl		Gh		Ln		*D* value
		*X*(*μ*)	*W*_*j*_	*X*(*μ*)	*W*_*j*_	*X*(*μ*)	*W*_*j*_	*X*(*μ*)	*W*_*j*_	*X*(*μ*)	*W*_*j*_	*X*(*μ*)	*W*_*j*_	*X*(*μ*)	*W*_*j*_	*X*(*μ*)	*W*_*j*_	*X*(*μ*)	*W*_*j*_	*X*(*μ*)	*W*_*j*_	*X*(*μ*)	*W*_*j*_	*X*(*μ*)	*W*_*j*_	
*Els*	10	0.06	0.03	0.61	0.04	0.30	0.02	0.11	0.02	0.23	0.04	0.02	0.00	0.15	0.01	0.47	0.00	0.32	0.02	0.14	0.02	0.34	0.02	0.30	0.02	3.79
	20	0.17	0.02	0.34	0.01	0.44	0.01	0.19	0.01	0.37	0.02	0.04	0.00	0.16	0.01	0.54	0.01	0.33	0.01	0.06	0.02	0.39	0.01	0.30	0.02	
	30	0.25	0.01	0.14	0.01	0.56	0.02	0.42	0.02	0.49	0.01	0.15	0.01	0.18	0.00	0.60	0.02	0.57	0.03	0.17	0.02	0.64	0.03	0.63	0.02	
	40	0.33	0.00	0.10	0.02	0.73	0.03	0.59	0.04	0.65	0.01	0.34	0.03	0.26	0.03	0.69	0.03	0.69	0.04	0.45	0.04	0.70	0.04	0.70	0.02	
	50	0.46	0.01	0.06	0.02	0.86	0.05	0.65	0.04	0.77	0.02	0.89	0.07	0.49	0.10	0.87	0.05	0.64	0.03	0.36	0.03	0.67	0.03	0.77	0.02	
*Elt*	10	0.14	0.02	0.56	0.04	0.21	0.03	0.19	0.02	0.41	0.02	0.51	0.04	0.06	0.04	0.12	0.04	0.19	0.03	0.12	0.02	0.19	0.02	0.20	0.02	1.50
	20	0.29	0.01	0.29	0.01	0.30	0.02	0.20	0.02	0.57	0.01	0.36	0.03	0.11	0.02	0.20	0.03	0.27	0.01	0.19	0.01	0.27	0.01	0.17	0.02	
	30	0.39	0.01	0.17	0.01	0.37	0.01	0.24	0.01	0.71	0.02	0.29	0.02	0.12	0.02	0.25	0.02	0.26	0.01	0.17	0.01	0.27	0.01	0.27	0.02	
	40	0.56	0.03	0.15	0.01	0.31	0.02	0.19	0.02	0.59	0.03	0.17	0.01	0.12	0.02	0.34	0.01	0.21	0.02	0.19	0.01	0.20	0.02	0.17	0.02	
	50	0.80	0.05	0.13	0.01	0.41	0.01	0.30	0.01	0.78	0.03	0.07	0.01	0.13	0.02	0.40	0.01	0.32	0.01	0.25	0.01	0.28	0.01	0.20	0.02	

## 4 Discussion

Cadmium is very mobile in plant tissues [32] and has toxic effects on plant-fruit germination and growth [33]. The fruit germination rates is a direct result of the growth and reproduction of plants and is the determinant of the *tangutorum* existence of plants in a certain area. The fruits germination rate for the two native plants on the Qinghai–Tibet Plateau decreased with the increase in cadmium concentration, indicating that the cadmium-polluted environment decreased the germination rates for both. This view was supported by Sami [34]. They found that the growth of rapeseed seedlings was inhibited under cadmium stress, and melatonin synthesis may be a key detoxification pathway to alleviate this inhibition. Cadmium stress can significantly reduce the final germination rate for *E. sinosubmuticus* S.L. Chen, but it has little effect on *E. tangutorum* (Nevski) Hand.-Mazz. These results indicate that although the two species are gramineous plants with the same growth as the Qinghai–Tibet Plateau, there are some differences in cadmium tolerance that may be caused by biological differences between different species, and these biological differences are ultimately attributed to the differences at the molecular level. This is similar to the results of Garcia de la Torre et al. [35], who studied the differences in cadmium tolerance of different alfalfa varieties. In their study, they found that among 258 alfalfa varieties with cadmium tolerance, the plant material containing the selected cadmium tolerance genotype pi516929 (CdT) had significantly higher cadmium tolerance than the plant material containing the sensitive genotype pi660497 (Cds). Furthermore, cadmium stress had negative effects on the fruits vigor, average fruit germination time, and germination index for both plants, which was the same as *Rhus typhoid* fruit germination under cadmium stress [36].

Plant growth is one of the important indicators for evaluating cadmium toxicity [7]. Studies have shown that low-concentration heavy-metal stress can promote plant growth, while high-concentration stress can inhibit plant growth and even kill plants [2,37,38]. Root-related data are of great significance in studying plant abiotic stress responses, as they are the primary sign of plants’ exposure to soil stressors [39]. Under cadmium stress, the root lengths of the two native plants on the Qinghai–Tibet Plateau changed significantly and decreased with the increase in cadmium concentration, while the stem and leaf lengths of the two grass species changed in the same way. The total biomass of both plants was significantly inhibited by the cadmium, indicating that cadmium toxicity can reduce the biomass of both plants. This corroborates the finding that cadmium is biologically toxic to most plants [40].

Fruits germination is essential for seedling growth. The germination of fruits will affect the later growth and development of seedlings. Therefore, it is important to study the relationship between fruits germination and seedling growth [41]. In this study, the 50% germination time for fruits was negatively correlated with the GSI and the vigor index, and had a strong correlation but little correlation with the growth of plant fruits, indicating that the effect of cadmium toxicity on the germination of the two grass species was not related to the fruits. The fruit germination and vigor indexes are positively correlated with growth indices, such as total biomass, root biomass, leaf–stem biomass, root length, and leaf length of fruits growth, and the correlation coefficient is large, indicating that the vigor index can be used as a future research tool. Fruit germination is positively correlated with fruit growth, this result is consistent with the relationship between fruits germination and fruits growth under water stress in bitter gourd (*Momordica charantia*) described by Adhikari et al. [42]. In their study, the water absorption and germination rates of Palee bitter gourd seeds were the highest after 48 h irrigation, while the water absorption and germination rates of seeds without irrigation were the lowest. Similarly, the seedlings soaked for 48 h had the highest values, and the seeds soaked for 36 h had the highest values, followed by the seedlings soaked for 36 h. The seedlings in the control group had the shortest and lowest values. In addition, Masondo et al. [43] also found that phytohormones relieve the three-leaf plant *Ceratotheca triloba* (Bernh.) under extreme temperatures, drought, and salt stress to promote fruit germination and fruit growth.

The Qinghai–Tibet Plateau has the characteristics of a large diurnal temperature difference, a short plant phenological period, severe cold in winter, and strong radiation [44,45]. Grassland is a major vegetation type on the Qinghai–Tibet Plateau, accounting for 70% of the vegetation area [46]. It is the main food source for yak, Tibetan sheep, and wild herbivores, of which the former two are the principal food source for local herdsmen [47]. *Elymus sinosubmuticus* S.L. Chen and *E. tangutorum* (Nevski) Hand.-Mazz. are common grasslands on the Qinghai–Tibet Plateau. Under cadmium stress, the fruits germination rate for the two grass species is reduced, the fruits vigor weakened, the growth of the fruits (especially the root system) is hindered, and the risk of heavy-metal pollution in the eastern Qinghai–Tibet Plateau increases. Against the background of the gradual increase [22,48], the stability of the plateau ecosystem and the environment on which animals and humans depend will be destroyed, presenting a serious challenge to the green concept of harmonious coexistence and development between humans and nature.

In a mining-contaminated floodplain pasture in central Wales, UK, the uptake of Zn, Cu, and (especially) Pb by sheep varies seasonally from 0.1 to 44% or more of soil dry matter in different seasons, and the accumulation of heavy metals in sheep may exceed safe thresholds [49]. Caribou populations in Western Greenland are also affected by heavy-metal pollution, with significantly higher levels of aluminum, arsenic, cadmium, lead, mercury, selenium, and zinc in the wetter, lichen-rich areas of the south and higher levels of copper in the arid desert grasslands of the north, where pasture dominates [50]. In other research, the average ^137^Cs content recorded for heather (*Calluna*) peatland in a Finnish reindeer management area was 44 ± 27 Bq/kg dw for dry heather, 75 ± 59 Bq/kg dw for medium heather, and 219 ± 150 Bq/kg dw for peatland vegetation, indicating that the health of reindeer was also seriously threatened [51]. These studies suggest that, in the future, it will be necessary to conduct research on the physiological and biochemical responses and gene expressions of the two native *Elymus* under cadmium stress on the Qinghai–Tibet Plateau.

## 5 Conclusion

It was found that with the increase of cadmium concentration, fruits germination rate, final germination rate, fruits vigor, average germination time and GSI of the two plants could inhibit fruits germination, and at the same time, biomass and leaf number of the two plants could be reduced. By using a simple fruit germination test and measuring seedling growth index, we can quickly screen plant materials for cadmium pollution (other heavy metals and environmental pollutants), which is of great significance for the search of green environmental protection and low-cost phytoremediation materials worldwide.
